# Correction: Evaluation of the initial timing of infection control pharmacist-driven audit and monitoring of vancomycin therapy in patients with infectious diseases: A retrospective observational study

**DOI:** 10.1371/journal.pone.0345047

**Published:** 2026-03-13

**Authors:** Hideki Sugita, Natsumi Okada, Matoka Okamoto, Kazumasa Abe, Masae Sekido, Michiko Tanaka, Tatsuro Tamatukuri, Yuika Naito, Masayuki Yoshikawa, Eisuke Inoue, Hironori Tanaka

The fourth author’s name is spelled incorrectly. The correct name is: Kazumasa Abe. The correct citation is: Sugita H, Okada N, Okamoto M, Abe K, Sekido M, Tanaka M, et al. (2023) Evaluation of the initial timing of infection control pharmacist-driven audit and monitoring of vancomycin therapy in patients with infectious diseases: A retrospective observational study. PLoS ONE 18(8): e0291096. https://doi.org/10.1371/journal.pone.0291096

The Data Availability statement for this article is incorrect. The correct statement is: Concerning data availability, there are restrictions on disclosing individual patients’ information for ethical considerations based the recommendation of the Ethics Committee on Research Involving Human Subjects at Showa University. Due to the limited number of patients admitted to our hospital and the small population size at our research centre, only the option of pseudo-anonymization of personal information is available to us. As such, the data set cannot be made available. Contact address: Hideki Sugita (hsugita@cmed.showa-u.ac.jp), Hironori Tanaka (h-tanaka@cmed.showa-u.ac.jp), Matoka Okamoto (matoka.o@cmed.showa-u.ac.jp), Kazumasa Abe (k.abe@cmed.showa-u.ac.jp), Masae Skido (m-sekido@cmed.showa-u.ac.jp), Michiko Tanaka (t.michiko@cmed.showa-u.ac.jp), Tatsuro Tamatsukuri (t.tatsuro@cmed.showa-u.ac.jp), Yuika Naito (n-yuika@cmed.showa-u.ac.jp), Masayuki Yoshikawa (masayuki-y@cmed.showa-u.ac.jp), Natsumi Okada (okada.natsumi.r4@luke.ac.jp), Eisuke Inoue (eisuke.inoue@med.showa-u.ac.jp).
