# Supplementary figures and images for: Evaluation of the initial timing of infection control pharmacist-driven audit and monitoring of vancomycin therapy in patients with infectious diseases: A retrospective observational study
Source: PLoS One. 2023 Aug 31;18(8):e0291096. doi: 10.1371/journal.pone.0291096 (PMC10470910; doi:10.1371/journal.pone.0291096)

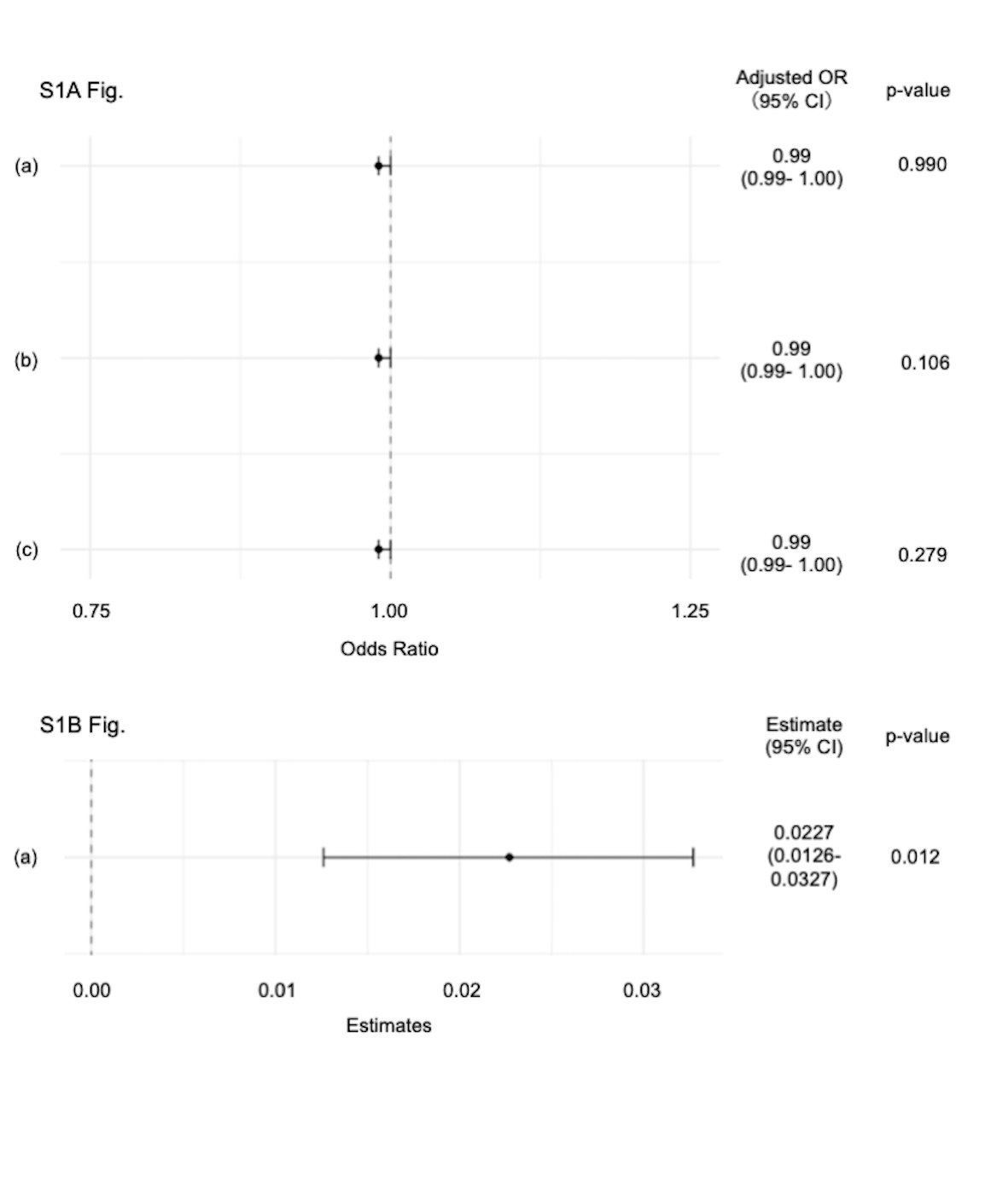

Supplement: S1 Fig — The main explanatory variables were the following continuous variables: the initial timing of the audit and monitoring intervention by the ICT pharmacists (h). A). (a) Maintenance of target trough concentration ranges for VCM during treatment duration, (b) Death within 30 days, (c) Implementation of de-escalation. Total number of patients was 638. No. of patients with events (%): (a) 307 (48.1), (b) 90 (14.1), (c) 206 (32.3). B). (a) Number of days of VCM administration. The average (SD) of number of days of VCM administration was 11.3 (7.4). (a) The odds ratios derived from the multivariable logistic regression or the estimates from the multiple linear regression were adjusted for age, sex, weight, eCCr, creatinine, AST, ALT, albumin, comorbidities (diabetes, cardiovascular diseases, renal failure, hypertension, and dyslipidaemia), immunosuppressant use, ICU admission, vancomycin loading dose, concomitant drug use (aminoglycoside, NSAIDs, piperacillin-tazobactam), and interventions of clinical pharmacists in charge of the wards. (b) The odds ratios derived from the multivariable logistic regression were adjusted for age, sex, eCCr, albumin, comorbidities (cardiovascular diseases), ICU admission, the loading dose of vancomycin, and interventions of clinical pharmacists in charge of the wards. (c) The odds ratios derived from the multivariable logistic regression were adjusted for age, sex, comorbidities (diabetes, cancer, cardiovascular diseases, COPD, hepatic disease, renal failure, hypertension, and dyslipidaemia), immunosuppressant use, ICU admission, and interventions of clinical pharmacists in charge of the wards. ICT, infection control team; OR, odds ratio; 95% CI, 95% confidence interval; SD, standard deviation; SE, standard error; VCM, vancomycin; eCCr, estimate creatinine clearance; AST, aspartate aminotransferase, ALT, alanine aminotransferase; ICU, intensive care unit; NSAIDs, non-steroidal anti-inflammatory agents; COPD, chronic obstructive pulmonary dis [file pone.0291096.s006.tiff]
