# Supplementary material for: Evaluation of the initial timing of infection control pharmacist-driven audit and monitoring of vancomycin therapy in patients with infectious diseases: A retrospective observational study
Source: PLoS One. 2023 Aug 31;18(8):e0291096. doi: 10.1371/journal.pone.0291096 (PMC10470910; doi:10.1371/journal.pone.0291096)
Supplement: S1 Checklist — (DOCX) [file pone.0291096.s007.docx]

STROBE Statement—checklist of items that should be included in reports of observational studies

|  | Item No. | Recommendation | Page  No. | Relevant text from manuscript |
| --- | --- | --- | --- | --- |
| **Title and abstract** | 1 | (*a*) Indicate the study’s design with a commonly used term in the title or the abstract | 1 | A retrospective observational study |
|  |  | (*b*) Provide in the abstract an informative and balanced summary of what was done and what was found | 2, 3 | To evaluate the impact of the initial timing of the audit and monitoring of vancomycin… |
| Introduction | | | |  |
| Background/rationale | 2 | Explain the scientific background and rationale for the investigation being reported | 3-5 | Infection prevention and control (IPC) is essential for patient safety and quality of care… |
| Objectives | 3 | State specific objectives, including any prespecified hypotheses | 5, 6 | However, no reports have examined the appropriate use of antimicrobial drugs… |
| Methods | | | |  |
| Study design | 4 | Present key elements of study design early in the paper | 6 | This retrospective observational study was conducted at an 815-bed university hospital in… |
| Setting | 5 | Describe the setting, locations, and relevant dates, including periods of recruitment, exposure, follow-up, and data collection | 6 | This retrospective observational study was conducted at an 815-bed university hospital in… |
| Participants | 6 | (*a*) *Cohort study*—Give the eligibility criteria, and the sources and methods of selection of participants. Describe methods of follow-up  *Case-control study*—Give the eligibility criteria, and the sources and methods of case ascertainment and control selection. Give the rationale for the choice of cases and controls  *Cross-sectional study*—Give the eligibility criteria, and the sources and methods of selection of participants | 7 | The eligibility criteria were hospitalised patients who had developed or were suspected… |
|  |  | (*b*) *Cohort study*—For matched studies, give matching criteria and number of exposed and unexposed  *Case-control study*—For matched studies, give matching criteria and the number of controls per case | N/A |  |
| Variables | 7 | Clearly define all outcomes, exposures, predictors, potential confounders, and effect modifiers. Give diagnostic criteria, if applicable | 8, 9 | The primary outcome was the continued achievement of optimal blood vancomycin… |
| Data sources/ measurement | 8* | For each variable of interest, give sources of data and details of methods of assessment (measurement). Describe comparability of assessment methods if there is more than one group | 8, 9 | The primary outcome was the continued achievement of…  The initial timing of the audit and monitoring intervention… |
| Bias | 9 | Describe any efforts to address potential sources of bias | 10, 11 | Multivariable logistic regression analysis for the primary…  Confounders that might alter the pharmacokinetics and… |
| Study size | 10 | Explain how the study size was arrived at | 11 | No previous studies have examined the effect of time on the auditing and monitoring… |

Continued on next page

| Quantitative variables | 11 | Explain how quantitative variables were handled in the analyses. If applicable, describe which groupings were chosen and why | 9 | The initial timing of the intervention was stratified into four exploratory groups based on… |
| --- | --- | --- | --- | --- |
| Statistical methods | 12 | (*a*) Describe all statistical methods, including those used to control for confounding | 10, 11 | Regarding the participants’ baseline characteristics, we aggregated the median, interquartile range (IQR)… |
|  |  | (*b*) Describe any methods used to examine subgroups and interactions | 10, 11 | Subgroup analyses were performed stratified by population: paediatric patients, ICU patients, non-ICU… |
|  |  | (*c*) Explain how missing data were addressed | 10, 11 | Missing values were imputed using the last-observation-carried-forward method. Furthermore… |
|  |  | (*d*) *Cohort study*—If applicable, explain how loss to follow-up was addressed  *Case-control study*—If applicable, explain how matching of cases and controls was addressed  *Cross-sectional study*—If applicable, describe analytical methods taking account of sampling strategy | N/A |  |
|  |  | (*e*) Describe any sensitivity analyses | 10, 11 | Missing values were imputed using the last-observation-carried-forward method. Furthermore… |
| Results | | | | |
| Participants | 13* | (a) Report numbers of individuals at each stage of study—eg numbers potentially eligible, examined for eligibility, confirmed eligible, included in the study, completing follow-up, and analysed | 12 | From the data on ICT practice during the study period, 956 patients were eligible…  Indicated in Figure 1. |
|  |  | (b) Give reasons for non-participation at each stage | 12 | Indicated in Figure 1 |
|  |  | (c) Consider use of a flow diagram | 12 | Indicated in Figure 1 |
| Descriptive data | 14* | (a) Give characteristics of study participants (eg demographic, clinical, social) and information on exposures and potential confounders | 13-17 | The baseline characteristics are summarised in Table 1. The median age of the patients was 69 years…  Indicated in Table 1. |
|  |  | (b) Indicate number of participants with missing data for each variable of interest | 16 | Indicated in Table 1. |
|  |  | (c) *Cohort study*—Summarise follow-up time (eg, average and total amount) | 17 | The median follow-up period for data collection was 30 days (IQR 30, 30). |
| Outcome data | 15* | *Cohort study*—Report numbers of outcome events or summary measures over time | 20, 21, 25-27 | Table 3, 4 |
|  |  | *Case-control study—*Report numbers in each exposure category, or summary measures of exposure | N/A |  |
|  |  | *Cross-sectional study—*Report numbers of outcome events or summary measures | N/A |  |
| Main results | 16 | (*a*) Give unadjusted estimates and, if applicable, confounder-adjusted estimates and their precision (eg, 95% confidence interval). Make clear which confounders were adjusted for and why they were included | 19-22 | Table 3 |
|  |  | (*b*) Report category boundaries when continuous variables were categorized | 20-22 | Table 3 |
|  |  | (*c*) If relevant, consider translating estimates of relative risk into absolute risk for a meaningful time period | N/A |  |

Continued on next page

| Other analyses | 17 | Report other analyses done—eg analyses of subgroups and interactions, and sensitivity analyses | 23-28, 39, 40 | Indicated in Table 4, S3 Table. |
| --- | --- | --- | --- | --- |
| Discussion | | | | |
| Key results | 18 | Summarise key results with reference to study objectives | 28 | We evaluated the impact of a retrospective audit and feedback targeting the anti-MRSA agent… |
| Limitations | 19 | Discuss limitations of the study, taking into account sources of potential bias or imprecision. Discuss both direction and magnitude of any potential bias | 31, 32 | This study had some limitations. This single-centre study was conducted in a university hospital… |
| Interpretation | 20 | Give a cautious overall interpretation of results considering objectives, limitations, multiplicity of analyses, results from similar studies, and other relevant evidence | 28-31 | There was no association between the period from initial vancomycin prescription to the audit and… |
| Generalisability | 21 | Discuss the generalisability (external validity) of the study results | 31 | Generalisations based on this study are limited. To generalise our results… |
| Other information | |  | | |
| Funding | 22 | Give the source of funding and the role of the funders for the present study and, if applicable, for the original study on which the present article is based | 33 | This work was supported by JSPS KAKENHI Grant-in-Aid for Early-Career Scientists, grant number JP20K18942. |

*Give information separately for cases and controls in case-control studies and, if applicable, for exposed and unexposed groups in cohort and cross-sectional studies.

**Note:** An Explanation and Elaboration article discusses each checklist item and gives methodological background and published examples of transparent reporting. The STROBE checklist is best used in conjunction with this article (freely available on the Web sites of PLoS Medicine at http://www.plosmedicine.org/, Annals of Internal Medicine at http://www.annals.org/, and Epidemiology at http://www.epidem.com/). Information on the STROBE Initiative is available at www.strobe-statement.org.
